# Acidosis, or Acetonæmia

**Published:** 1908-10-03

**Authors:** 


### Pathology.

### ACIDOSIS, OR ACETONÆMIA.

Recent studies in physiological and in pathological bio-chemistry have thrown a great deal of light upon the metabolic processes which lead to the presence of acetone in the blood and urine; and much that is of very real value in practical medicine has been discovered in connection with acidosis.

From a prognostic point of view, diabetic patients may be divided into two main classes: those who are passing diacetic acid and acetone along with the glucose in their urine, and those who are passing glucose but no acetone or diacetic acid. There is little likelihood of a diabetic patient passing into coma so long as the urine is free from acidosis products. Since this discovery was made, a great many other facts have been determined in regard acetonæmia.

It may be asked by those who have not been in a position to follow the literature of the subject, what acidosis, acetonæmia, and acetonuria are, and how it comes about that the terms can be used as almost synonymous, or at least interchangeable. The answer necessitates a brief reference to some errors of metabolism that occur under certain conditions. When fats, carbohydrates, and proteids are katabolised in the body the carbon ultimately assumes one of two forms: $CO_2$, which is exhaled from the lungs; and nitrogenous compounds containing carbon, such as urea, uric acid, and the like, which are mainly excreted in the urine. Under normal conditions, there is hardly any elimination of carbon uncombined with nitrogen except in the form of $CO_2$. Under certain abnormal circumstances, however, carbon may be eliminated in the urine uncombined with nitrogen. It may, for instance, be excreted unkatabolised—as glucose; and it may be eliminated after katabolism of an abnormal kind—as acetone and its allies. It is when the latter abnormal substances are present in the blood that one speaks of "acetonæmia"; when they are detected in the urine one speaks of "acetonuria"; and acetonæmia is presumable if acetonuria can be demonstrated; whilst the patient displaying the erroneous form of metabolism from which acetonuria results is said to be the subject of "acidosis."

Acetone is not the only product of acidosis known; it is the last of a series of substances, each more oxidised than the one before it, and of the series at least two others are well understood; these are -oxybutyric acid and aceto-acetic (diacetic) acid.

All these three substances frequently occur in the blood and in the urine together; but for the sake of convenience one speaks only of acetonæmia and acetonuria.

There has been a great deal of discussion as to what is the source of these substances in the body. It is generally conceded that they are not directly derived from any of the carbohydrate ingested by the patient; but opinions differ as to whether proteid or fat is their source. Schwarz, who has investigated the matter very exhaustively, blames the fat, stating his belief that abnormal destruction of body fat is the cause of the excretion of acetone bodies in various conditions, such as malignant cachexia, acute infectious fevers, and conditions in which there is severe upset of the gastro-intestinal functions. In support of this view much stress has been laid upon the fact that a perfectly healthy man passes acetone in his urine if he refrains from eating for a considerable time. Starvation seems undoubtedly to be an important cause of acetonuria, but it does not follow that the acetone is derived from the patient's fat. It may come from his proteid, which also breaks down during starvation. Those who hold that abnormal proteid metabolism is at the root of acetonuria lay stress upon the occurrence of $\beta$-amido-oxybutyric acid under similar circumstances—the "amido" portion of this acid being evidence of its derivation from nitrogenous katabolism.

### Tests for Acetone in the Urine.

There are two tests for acetone in urine—namely (1) the iodoform test, and (2) the sodium nitroprusside test. Of these the former is the more frequently quoted, but the latter is the more easy of application in practice. The main objection to the iodoform reaction is the necessity for concentrating the acetone by distillation of the urine before the test can be applied with any degree of certainty; but after distillation the iodoform test is probably the better of the two. A solution of iodine in potassium iodide is added drop by drop to, say, 10 c.c. of the urinary distillate in a test tube, the latter being warmed gently meanwhile; if acetone is present, iodoform is formed, and it is recognised both by its smell and by the appearance of the yellow crystals that will be obtainable from the solution.

The sodium nitro-prusside test, on the other hand, is a colour reaction, and serves to detect acetone with sufficient accuracy for clinical purposes even without preliminary distillation of the urine. A good way of performing it is to have two test tubes, one of which is quarter filled with the suspected urine, whilst an equal quantity of a normal urine is poured into the other. To both tubes one now adds a few drops of strong caustic soda solution; there may be no change in the contents of either test tube, or there may be a milky turbidity due to the precipitation of phosphates. To each is now added, drop by drop, some saturated solution of sodium nitro-prusside, until a good red colour is produced in the upper part of the fluid. It is well not to shake up the tubes, so that the lower portions of the contents of each may serve as colour-comparisons. The red colour will appear in both the normal urine and in that which contains acetone; it is produced by the creatinin in healthy urine. Upon now adding glacial acetic acid drop by drop to each test tube, however, the red colour in the normal urine will be discharged, whereas in the urine which contains acetone the red colour, far from being discharged, becomes deeper and deeper, until the fluid is of a rich red. This reaction is very easy indeed to obtain, and it is very striking.

### Test for Diacetic Acid in Urine.

It is possible for a urine to contain much diacetic acid and little or no acetone, hence it is always advisable to test for diacetic acid as well as for acetone. Moreover, if both reactions are positive the diagnosis of acetonæmia is more certain than if acetone alone is tested for; and the absence of acetonæmia is better established when the urine is known to contain neither acetone nor diacetic acid. Liquor ferri perchloridi (B.P.) should be added drop by drop to a test tube half-filled with the urine. The first few drops of the solution cause a dense white precipitate of phosphates; later, as more and more is added drop by drop, a red, reddish brown, or reddish purple colour is produced if diacetic acid is present. The tint is very variable, and according as there is much or little diacetic acid in the specimen it may be either quite pale red on the one hand or deep Burgundy colour upon the other. A similar, but much more purple, colour is given by the urine of a patient who is taking salicylic acid, or one of its compounds, such as sodium salicylate, or aspirin.

### Conditions under which Acetonæmia Occurs.

It is not certain whether acetone, diacetic acid, and $\beta$-oxybutyric acid are themselves the cause of diabetic coma, or whether they are only signals of the acidosis which kills the patient. The clinical significance of acetonuria is much the same in either case, and probably the acetone deserves more attention than does the sugar. A patient who is passing much sugar, but no acetone, is in a much safer condition than is one who is passing a great deal less sugar but considerable quantities of acetone. Treatment which minimises the amount of acetone and allied substances in the urine is more likely to do good than that which is solely directed to diminishing the degree of glycosuria. Acetonuria may be decreasing when the glycosuria is increasing, and *vice versa*.

It is not only in diabetics, however, that acidosis and acetonuria occur. Reference has already been made to starvation as a cause for them in absolutely healthy persons. Similarly, patients whose illness causes more or less starvation are likely to suffer from acidosis and acetonuria. Acetone may be found in the urine in cases, for example, of malignant cachexia, chronic gastric ulcer, gastrectasis, high fevers, pneumonia, acute and chronic intestinal obstruction, and other acute abdominal conditions. such as appendicitis. In many such cases the acidosis is temporary and unimportant; in many others the acidosis, though not temporary, is of very minor importance. In some cases, however, the symptoms of acetonæmia and acidosis are superimposed upon those due to the causal ailment, and treatment is required for the acidosis itself. The primary and the secondary symptoms are apt to be so commingled that it is difficult to say exactly what the symptoms of acidosis are; but evidence is rapidly accumulating to show that delayed anæsthetic poisoning, infantile convulsions, the prostration of severe diarrhœa and vomiting of children, the uncontrollable and even fatal vomiting of pregnancy, and some of the severe after effects of prolonged surgical operations, are all instances of acidosis, and that in all of them acetonuria is to be found if it is looked for. In diabetes dulness and apathy may increase as the condition progresses, and in severe cases end in coma. In other patients restlessness and delirium take the place of dulness and apathy; and convulsions are not uncommon if the patients are children. Vomiting if it occurs is severe, being at times almost or quite uncontrollable. The patent's mental state may be such that meningitis is feared.

### The Treatment of Acidosis.

It is not yet possible to adopt active measures to stop the erroneous metabolism which gives rise to acetonæmia, because there is still doubt as to where the metabolic fault lies. Something can be done, however, to prevent the occurrence of acidosis, and much can be accomplished in counteracting the effects of acidosis when it has occurred. The prophylactic measures in diabetes are well known—avoidance of too sudden a curtailment of the carbohydrate food, and of undue physical fatigue or mental overstrain. Starvation being a cause of acetonuria, it is clearly unwise to go on starving gastric and other cases which have acetone in their urines, and so on.

The curative measures resolve themselves into the administration of alkalies in large doses, to neutralise the acid products in the blood. Neither the blood nor the urine become unduly acid when acidosis is taking place—a matter which may seem paradoxical; but in order to neutralise the diacetic and $\beta$-oxybutyric and other acids as they are formed, a considerable quantity of the nitrogen which should appear in the urine as urea becomes excreted in the form of combined ammonia. If it were not so difficult to estimate the latter, a very fair measure of the degree of acidosis occurring in any given patient would be afforded by the amount of this combined ammonia. Alkalies are not given, therefore, with the idea of making the blood either less acid or more alkaline; they are given in order to relieve the strain upon the patient's powers of ammonia-compensation for acidosis. The giving of alkalies has produced wonderfully good results in some cases. One has seen lives saved by gastric lavage with weak sodium bicarbonate solution for uncontrollable vomiting after long operations under anæsthetics. One has seen diabetic patients brought round from coma, and others saved from threatening coma, when big doses of sodium bicarbonate were given. Much of the good attributable to bismuth in gastric cases may be due to the salt most frequently prescribed being the oxycarbonate. It is clear that different conditions require the alkali in different quantities. For diabetes with impending coma as much as 100 grams of sodium bicarbonate per diem has been given by the mouth. In urgent cases the hypodermic method may be resorted to, the strength employed being 1 of bicarbonate of soda in 300 of water. It may be given rectally when oral administration is not tolerated. The main thing is to recognise acetonæmia when it is present, by testing for acetonuria as a routine measure.

